# Supplementary material for: The Microbiome of Peri-Implantitis: A Systematic Review and Meta-Analysis
Source: Microorganisms. 2020 May 1;8(5):661. doi: 10.3390/microorganisms8050661 (PMC7284896; doi:10.3390/microorganisms8050661)
Supplement: Supplementary file 1 [file microorganisms-08-00661-s001.pdf]

Supplementary Material

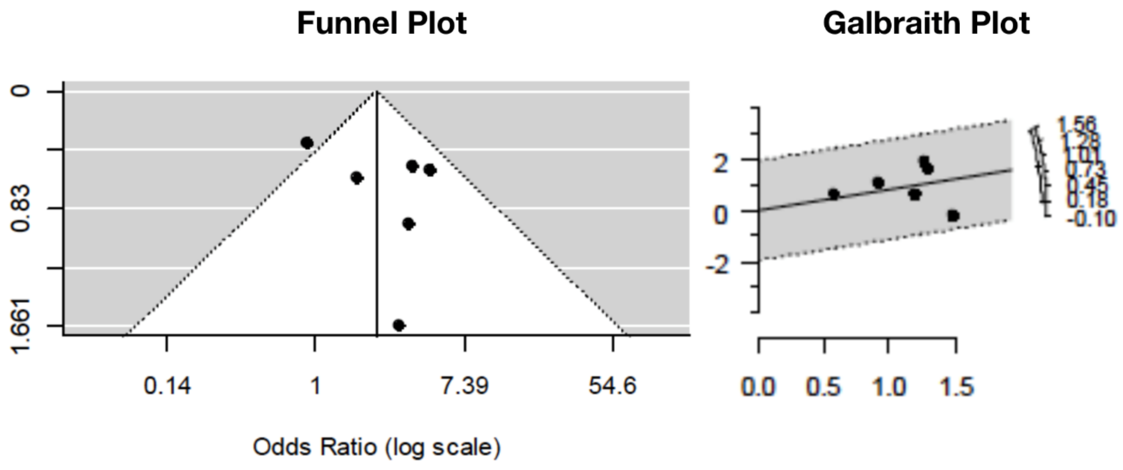

Figure S1. funnel and Galbraith plot for the presence of P. intermedia in the different studies.
